# Bacterial Communities in Pigmented Biofilms Formed on the Sandstone Bas-Relief Walls of the Bayon Temple, Angkor Thom, Cambodia

**DOI:** 10.1264/jsme2.ME13033

**Published:** 2013-12-13

**Authors:** Asako Kusumi, Xianshu Li, Yu Osuga, Arata Kawashima, Ji-Dong Gu, Masao Nasu, Yoko Katayama

**Affiliations:** 1 United Graduate School of Agricultural Science, Tokyo University of Agriculture and Technology, 3–5–8 Saiwai-cho, Fuchu-shi, Tokyo 183–8509, Japan; 2 Graduate School of Agriculture, Tokyo University of Agriculture and Technology, 3–5–8 Saiwai-cho, Fuchu-shi, Tokyo 183–8509, Japan; 3 School of Biological Sciences, The University of Hong Kong, Pokfulam Road, Hong Kong SAR, People’s Republic of China; 4 Advanced Pharmaco-science, Graduate School of Pharmaceutical Sciences, Osaka University, 1–6 Yamada-oka, Suita, Osaka 565–0871, Japan

**Keywords:** bacterial community, denaturing gradient gel electrophoresis, non-destructive sampling, cultural heritage, biodeterioration

## Abstract

The Bayon temple in Angkor Thom, Cambodia has shown serious deterioration and is subject to the formation of various pigmented biofilms. Because biofilms are damaging the bas-reliefs, low reliefs engraved on the surface of sandstone, information about the microbial community within them is indispensable to control biofilm colonization. PCR-denaturing gradient gel electrophoresis (DGGE) analysis of biofilm samples from the pigmented sandstone surfaces showed that the bacterial community members in the biofilms differed clearly from those in the air and had low sequence similarity to database sequences. Non-destructive sampling of biofilm revealed novel bacterial groups of predominantly *Rubrobacter* in salmon pink biofilm, *Cyanobacteria* in chrome green biofilm, *Cyanobacteria* and *Chloroflexi* in signal violet biofilm, *Chloroflexi* in black gray biofilm, and *Deinococcus-Thermus*, *Cyanobacteria*, and *Rubrobacter* in blue green biofilm. Serial peeling-off of a thick biofilm by layers with adhesive sheets revealed a stratified structure: the blue–green biofilm, around which there was serious deterioration, was very rich in *Cyanobacteria* near the surface and *Chloroflexi* in deep layer below. Nitrate ion concentrations were high in the blue–green biofilm. The characteristic distribution of bacteria at different biofilm depths provides valuable information on not only the biofilm formation process but also the sandstone weathering process in the tropics.

A biofilm is an aggregation of microbial cells and extracellular polymer substances (EPSs) adhering to a solid substratum surface. Biofilms formed on stone monuments accelerate rock decay through physicochemical processes, namely the excretion of water-absorbing EPSs that contain surface-tension-reducing compounds and the uptake of capillary water followed by an increase in water-holding capacity (46). Biodeterioration caused by biofilm formation has been reported in many stone structures of cultural heritage (41, 47). In the Angkor monuments of Cambodia, there have been analyses of microorganisms in fresh and old biofilms by PCR amplification and clone libraries (21) and observations of colonizing cyanobacteria in green–brown and brown biofilms (9). Although the walls of the Bayon temple at Angkor Thom are covered with biofilms pigmented with various colors, there has been no comprehensive study on the bacterial communities in these biofilms.

Cambodia’s Angkor monuments are revered by the local people and have attracted visitors from around the world. In particular, the Bayon temple in the center of the historical city of Angkor Thom in Siem Reap is one of these great monuments. Its foundations are of laterite and its surface is of sandstone. On the walls of the inner and outer galleries that surround the central towers are engraved unfolding picture stories of Hindu myths and the life of the Khmer people as bas-reliefs. The temple has been deteriorating as a result of various factors, including water intrusion, mineral crystallization and microbial action, as is the case with many other historical stone monuments (41, 47). Exfoliate weathering, the flaking-off of fairly large and flat pieces of rock (14), has been observed at the base of sandstone pillars in Angkor Wat, where water intrusion is apparent. Previous studies showed that sulfur-oxidizing microorganisms play an important role in the decay of sandstone (23); sulfur-oxidizing *Fusarium* sp. and *Mycobacterium* spp. were isolated and their sulfur oxidation via chemolithoautotrophic process has been elucidated (19, 24).

In contrast, at the Bayon temple, biofilm formation on the sandstone walls is a more serious problem. Various pigmented biofilms are affecting not only the temple’s aesthetic appearance but also the integrity of its materials underneath (10, 27, 47) and the valuable bas-reliefs. Information about the structure of the microbial communities in the biofilms is therefore indispensable for understanding the microbial community involved and also controlling biofilm colonization of the historic bas-reliefs. To characterize the microorganisms in biofilms, we first compared bacteria in the air collected at the temple with bacteria found in the biofilms. The characteristics of the bacterial communities in variously pigmented biofilms and the stratified structure of bacteria in the biofilm were compared by using 16S rRNA-based PCR-denaturing gradient gel electrophoresis (DGGE) analyses among the bacterial communities after non-destructive collection of biofilm samples onto adhesive sheets. In addition, nitrate ions in the biofilms were also quantified as an indicator of microbial metabolism inside them due to ammonia/ammonium oxidation.

## Materials and Methods

### Sampling sites

The Bayon temple was built in the late 12th century. The climate of the area is governed by two monsoons: the northeastern monsoon resulting in dryness from November to April, and the southwestern monsoon brings the rainy season from May to October (25). Annual rainfall at the Angkor site is 1,300 to 1,500 mm (17) and the average temperature is 25°C (16). In the inner gallery of the Bayon the monthly mean temperature is between 25 and 30°C throughout the year, and the monthly mean relative humidity is between 60% and 80% in the dry season and 80% and 90% in the rainy season (39).

**Fig. 1:**
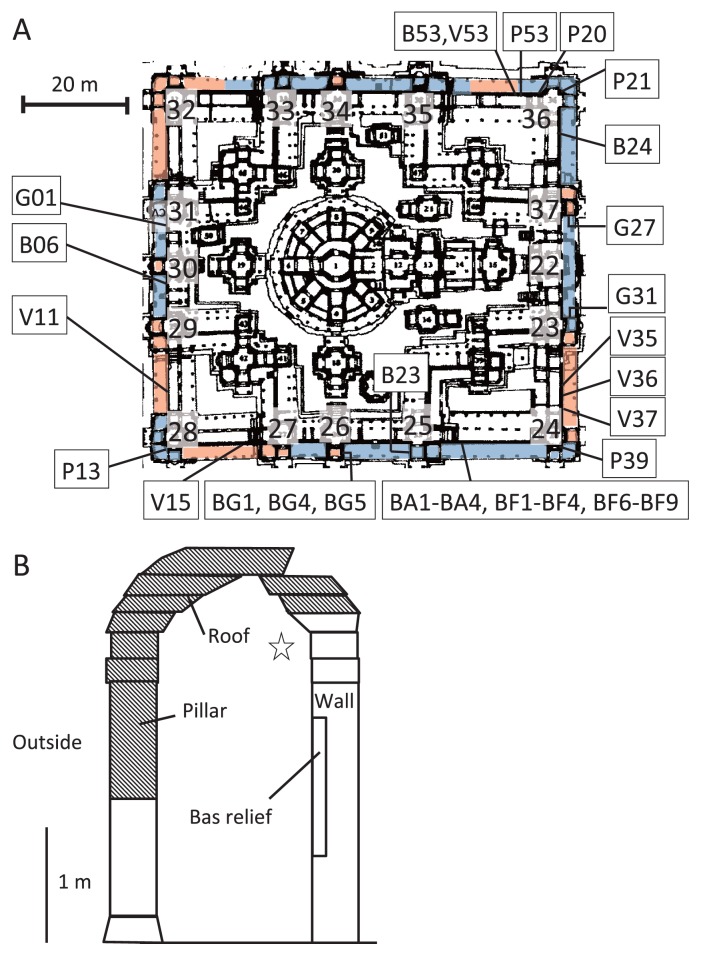
**A.** Map of the inner gallery of the Bayon, Angkor Thom, showing locations where biofilm and airborne microorganism samples were collected. Sample names are prefixed by the abbreviation of each biofilm color; sample identifications and biofilm characteristics are given in Table 1. The number next to the tower is the tower number. Blue area shows the area where the roof remained. Orange area shows the area where the roof was partly or completely missing. **B.** Side view of the inner gallery, which consisted of a sandstone wall on which bas-reliefs were engraved and a roof supported by pillars. Hatched areas show the parts of the pillars or roof that were partly or completely collapsed or missing. The area from which airborne bacterial samples were collected is shown by an asterisk.

Airborne microbes and biofilms colonizing on the sandstone wall of sampling points (BA1 to BA4, BF1 to BF4, and BF6 to BF9, Fig. 1A) where the roof still stands were collected in September 2009. All other biofilm samples (Fig. 1A) were collected in September 2008, September 2009 or September 2011. Airborne microbes were collected onto an autoclaved polycarbonate membrane filter (pore size, 0.20 μm; disk diameter, 47 mm; Advantec, Tokyo, Japan), which was mounted in an open-face polycarbonate filter holder (Advantec) disinfected with 70% (v/v) ethanol. The filter holder was placed 2.5 m above the floor and 20 cm from the wall and connected to a diaphragm-type dry vacuum pump (model DA-30S; Ulvac Kiko, Kanagawa, Japan). Each air sample (1.4×10^4^ L) was passed through the filter at 5 L min^−1^; this rate was controlled with a DMQ-V digital mass-flow controller (model MQV0050; Yamatake, Tokyo, Japan). Four samples (BA1 to BA4) were collected (once every 48 hours) over an 8-day period. After each sampling, the filters were put back into sterilized filter cases. To compare the bacterial communities of the air samples and the biofilms formed on the sandstone wall, biofilm samples were collected from the sandstone wall near the filter holder. Eight samples (BF1 to BF4 and BF6 to BF9) were collected by using adhesive sheets sterilized by gamma irradiation (adhesive area: 2.4×3.0 cm) (48). The colors of the biofilms sampled were brown for samples BF1, BF2, and BF9, brown and black for sample BF8, black for sample BF3, dark green for samples BF4 and BF6, and green for sample BF7. All samples were kept cold in an ice cooler in the field and stored at −20°C in a refrigerator of local office in Siem Reap, Cambodia, for a few days; they were then transported to our laboratory, where they were kept at −80°C until DNA extraction.

**Fig. 2:**
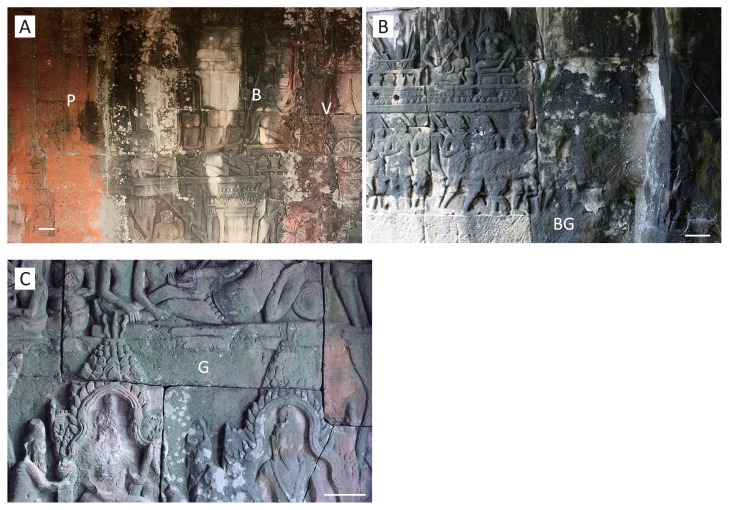
Various pigmented biofilms formed on the sandstone walls of the inner gallery of Bayon. Bar indicates 10 cm. **A.** Salmon pink (P), black–gray (B), and signal violet (V) biofilms on the north-facing wall on the north side. Samples P53, B53, and V53 were obtained from the positions indicated “P”, “B”, and “V”, respectively. **B.** Blue–green (BG) biofilm on the east-facing wall on the south side. Samples BG1, BG4, and BG5 were obtained from the position indicated “BG”, measuring 30 cm in circumference. Sample BG4 was used for analysis of bacterial stratified structure. **C.** Chrome green (G) biofilm on the south-facing wall of the east side. Sample G27 was obtained from the position indicated by “G”.

To compare the bacterial communities of the various pigmented biofilms, adhesive sheets were used to take microorganisms from sandstone surface for routine analysis at 21 locations in the inner gallery (see Fig. 1A and Table 1 for sample numbers); the biofilms were grouped according to the five major colors observed on the gallery walls (Fig. 2A–C). The designated colors were based on a color chart (RAL Classic, German Institute for Quality Assurance and Certification, Sankt Augustin, Germany) as follows: salmon pink (P), RAL 3022; chrome green (G), RAL 6020; signal violet (V), RAL 4008; black–gray (B), RAL 7021; and blue–green (BG), RAL 6004. Microorganisms in the BG biofilm were collected from the surface to the deepest layer by a series of peeling-off the biofilm with adhesive sheets (23 sheets) at the same sampling position to analyze the stratified bacterial structure (sampling site BG4, Fig. 2B). This biofilm was chosen because it appeared thickest and seemed to be causing more serious deterioration of the biofilm underneath.

To monitor the amounts of nitrate ion in the biofilms, sample No. 5 was taken from the wall near the BG; samples No. 1, 2, 9 and 10 were taken from the outer gallery where the roof was completely missing; samples No. 3, 4, 7 and 8 were taken from the inner gallery, where the roof was still present (Fig. 1B). The collected amount of biofilm from stone of no cultural importance was less than 1 g.

### DNA extraction and PCR amplification

Samples were subjected to DNA extraction with a FastDNA spin kit for soil (MP Biomedicals, Illkirch, France). For DNA extraction, we performed one experiment on each sample because a tiny amount of sample was taken from the wall. Instead of replicates, we checked many samples of the similar colored biofilms. The adhesive side of the sheet used to sample the biofilm was placed in a tube, facing inward. DNA was extracted according to the kit manufacturer’s specifications.

To profile the bacterial community by DGGE, the V1 to V3 region of the bacterial 16S rRNA gene was amplified with the GC8F primer set, which was designed to attach a GC-clamp (cgcccgccgcgccccgcgcccgtcccgccgcccccgcccg) to the 5′ terminal of 8F (50), and with 520R (22). The PCR mixture contained 10 μL of 5× Phusion HF Buffer (Finnzymes, Espoo, Finland), 1 μL of 10 mM dNTPs (200 μM each), 1 μL of each primer (0.5 μM each), 5 μL of 0.1% bovine serum albumin, 1 μL of dimethyl sulfoxide, 0.5 μL of Phusion DNA polymerase (Finnzymes), 1 μL of Milli-Q water, and 1 μL of template DNA (0.1 to 20 ng) in a total volume of 50 μL. PCR amplification was performed in a thermal cycler (TP600, TaKaRa PCR thermal cycler dice; TaKaRa Bio, Otsu, Japan). The amplification conditions were as follows: 98°C for 30 s (initial denaturation), followed by 30 cycles of 98°C for 5 s, 50.7°C for 15 s, and 72°C for 60 s, with a final extension step of 72°C for 10 min. The amplified PCR products were checked on 1.5% agarose to verify amplification of the target region and purified with a GFX PCR DNA and gel band purification kit (GE Healthcare Life Sciences, Tokyo, Japan); they were then quantified with a UV spectrophotometer.

### DGGE analysis

DGGE was performed as described by Muyzer *et al.* (29) using the DCode universal mutation detection system (Bio-Rad Laboratories, Hercules, CA, USA). DNA samples (150 ng) were loaded onto a 10% (w/v) polyacrylamide gel that contained a linear gradient of 30% to 70% denaturant (100% denaturant contained 7 M urea and 40% formamide). After electrophoresis at 60°C and 75 V for 14 h, the gel was soaked in SYBR Green I nucleic acid gel stain (Molecular Probes, Eugene, OR, USA) for 30 min, and then visualized and photographed under UV transillumination using a Printgraph (DT-20MP; Atto, Tokyo, Japan). DGGE marker I (Nippon Gene, Toyama, Japan) was used as a standard marker.

The relevant positions of each band on the DGGE gel were detected, and the peak intensities were converted to peak heights with CS Analyzer 3 (Atto). The bands were considered positive when the peak height as a percentage of the total peak height exceeded 5% in each lane in order to show the bacterial compositional characteristic more clearly. Bacterial compositions were calculated on the basis of the number of bands observed on the DGGE gels after analysis of the phylogenetic position as described below.

### Sequence analysis of DGGE bands

Bands in the DGGE gel were excised from the gels with a Gel Cutting Tip (Axygen, CA, USA) and suspended in 50 μL of sterilized TE buffer (10 mM Tris-HCl, 0.5 M EDTA; pH 8.0). In order to check whether the target bands were obtained exactly, we compared with the DGGE gel picture before and after excising the bands. The DNA recovered after freeze-thawing the suspension was amplified under the same conditions as described above, except that as the forward primer we used 8F instead of GC8F. The size and amount of the PCR products were checked by 1.5% agarose gel electrophoresis for 30 min at 100 V. Cleaned PCR products were sequenced with a BigDye Terminator v3.1 Cycle Sequencing Kit (Applied Biosystems, Carlsbad, CA, USA) by using the following reaction mixture: 0.5 μL of Ready Reaction Premix, 2 μL of 5×BigDye Sequencing Buffer, 0.32 μM of primer, cleaned PCR template, and Milli-Q water to a 10-μL total reaction volume. Amplification of cycle sequencing was as follows: 96°C for 1 min (initial denaturation) followed by 25 cycles of 96°C for 10 s, 50°C for 5 s, and 60°C for 4 min. Sequencing reactions were cleaned by ethanol precipitation, and sequencing was performed on an ABI 3130 Genetic Analyzer (Applied Biosystems). The closest relatives of the sequences of the DGGE bands were searched for in the GenBank database with a BLAST search program.

### Phylogenetic analysis of 16S rRNA gene sequence

Each DNA sequence was compared with the sequences available in the database by using BLASTN 2.2.26 (51) from the National Center for Biotechnology Information (http://blast.ncbi.nlm.nih.gov/Blast.cgi). Potential DNA chimeric structures were detected by separately determining the species affiliation and the query coverage of the terminal 5′ and 3′ structures of each sequence. All of the sequences and their closest relatives were then aligned, and a phylogenetic tree was constructed with MEGA 5 (44) using the neighbor-joining method (37) with gaps and missing data removed. The presence of chimeric sequences was checked by comparison with the sequences in the Chimera-Checked database using BLASTN (1) from Greengenes (7; http://greengenes.lbl.gov/cgi-bin/nph-index.cgi).

### Nitrate ion concentration in the biofilms

Biofilm samples collected in 2011 (0.1 g as wet weight) were suspended in 0.5 to 1.0 mL Milli-Q water and centrifuged at 5,000×*g* for 5 min at 4°C. The supernatant was used to measure pH with a compact pH meter (B-212; Horiba, Kyoto, Japan) and the concentration of nitrate ions with a compact NO_3_^−^ meter (B-343; Horiba). These measurements were performed at the local office in Siem Reap. For nitrate ion measurements, we performed one experiment on each sample because a tiny amount of sample was taken from the wall. Instead of replicates, we checked many samples of the similar colored biofilms.

### Nucleotide sequence accession numbers

All sequences of the 16S rRNA genes determined in this study have been submitted to the DNA Data Bank of Japan under accession numbers AB679757 to AB679829 and AB758608 to AB758622 (Tables S1 and S2).

## Results and Discussion

### Bacteria in wall biofilms and air

**Fig. 3:**
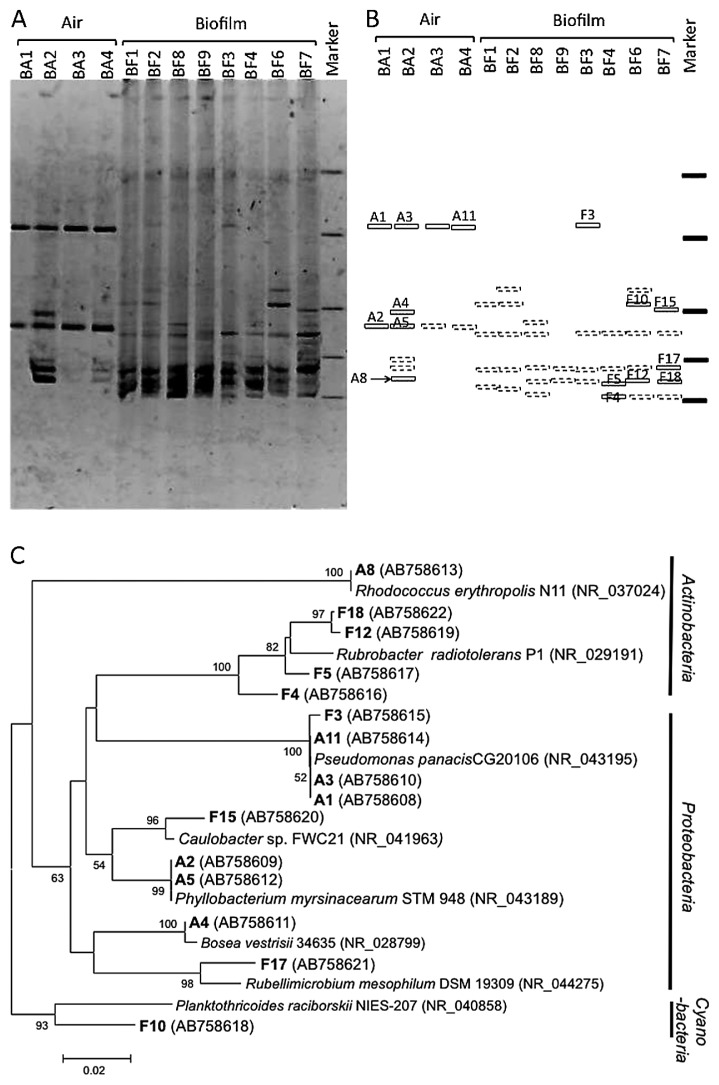
Comparison based on partial 16S rRNA gene sequencing of bacterial communities in air collected in the inner gallery and in biofilms formed on the sandstone wall close to the air-sampling location. **A.** Bacterial community fingerprints of air (BA) and biofilm (BF) samples by DGGE analysis. The partial 16S rRNA gene was amplified with primers GC8F and 520R. **B.** Schematic diagram of the DGGE band patterns in **A**. Labeled bands correspond to 16S rRNA gene sequence types described in **C** and Table S1. Broken lines are DGGE bands that were not sequenced. **C.** Phylogenetic relationships based on partial 16S rRNA gene sequences derived from air and biofilm samples by DGGE analysis. Sequences recovered in this study and corresponding to the band names in **B** are shown in bold type; “A” indicates bands derived from the air sample and “F” indicates bands derived from the biofilm sample. Numbers in parentheses are GenBank accession numbers. Neighbor-joining tree: bootstrap values based on 1,000 replicates are indicated for branches supported by 50% of trees. Scale bar represents 0.02 nucleotide changes per position.

Bacteria in the wall biofilm samples BF1 to BF4 and BF6 to BF9 and the ambient air were compared by using DGGE to characterize the microorganisms in biofilms formed on the stone surface. The DNA banding patterns of PCR-amplified 16S rRNA genes, and hence the bacterial community structures, of the biofilm samples clearly differed from those of the bacteria found in the air samples (Fig. 3A). Two major DNA bands that were observed at the same position on the DGGE gels from all biofilm samples were *Rubellimicrobium* (band F17) and *Rubrobacter* (bands F5, F12, and F18) (Fig. 3A and B, Table S1), indicating that the major bacteria found in the biofilm were *Proteobacteria* and *Actinobacteria* (Fig. 3B and C). Except for band F3 and F15, the bacterial DNA sequences found in the biofilm had low levels of identity (91% to 94%) to the sequences in the database (Table S1). As already reported at many other historical sites (26, 40, 43), including Angkor sites (9, 21), the major bacteria in the biofilms colonizing the stone surfaces were characterized by the presence of unknown bacterial populations that were suited to growth in this environment.

In contrast to the bacteria found in the biofilms, the DNAs of only two major bacterial genera prevailed in the air samples (Fig. 3A): bands A1, A3, and A11 were affiliated with *Pseudomonas panacis* CG20106 with 100% identity, and bands A2 and A5 were affiliated with *Phyllobacterium* spp. with 98% or 100% identity (Table S1). The numbers of bands in the biofilm samples were higher than in the air samples (*P*<0.05) indicated that the bacterial diversity in the biofilm was higher than in the air. There has been a focus on airborne microorganisms at historical sites (33, 36, 45) because the atmosphere is a major vehicle for microorganisms (31). However, our results showed that the bacteria in the biofilm were unique and that major airborne bacteria seemed unable to establish communities under the extreme environmental conditions of exposure to severe sunlight and wind on the stone surface.

### Bacterial communities in the pigmented biofilms

The P, G, and BG biofilms (Fig. 2) were frequently observed on the walls in places where the roof was still standing. The P biofilm was thin and dry. The BG biofilm was obviously thick (approx. >1 mm), comparatively wet, and easy to peel. The V biofilm was thin (approx. <1 mm) and was usually observed on the walls in places where the roof was missing (Fig. 1A). The stone surface under the BG biofilm exhibited exfoliation and serious deterioration. The G biofilm was comparatively thin (approx. <1 mm), hard, and difficult to peel off. The B biofilm was thin, easy to peel off, and was observed on the walls in places where the roof was either present or missing.

**Fig. 4:**
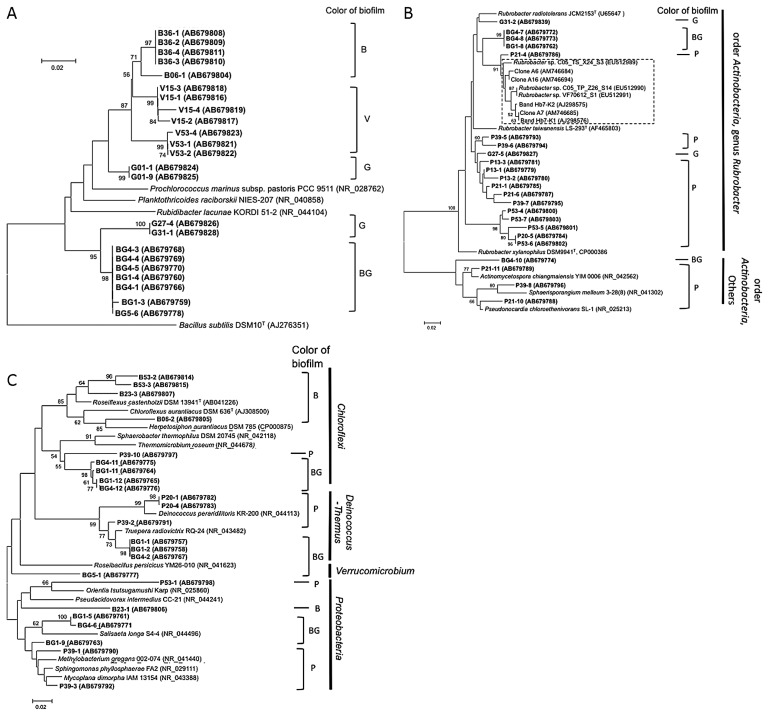
Phylogenetic relationships based on partial 16S rRNA gene sequences recovered from salmon pink (P), chrome green (G), signal violet (V), black–gray (B), and blue–green (BG) biofilms derived from sandstone walls at Bayon by DGGE analysis. Sequences recovered in this study are shown in bold type. Numbers in parentheses are GenBank accession numbers Neighbor-joining tree: bootstrap values based on 1,000 replicates are indicated for branches supported by 50% of trees. *Scale bar* represents 0.02 nucleotide changes per position. **A.** Phylogenetic affiliation of Cyanobacteria clade members. *Bacillus subtilis* DSM10^T^ was used as an outgroup. Dashed box indicates 16S r RNA sequences derived from rosy discoloration of European historical stone monuments (15, 20, 40). **B,** Phylogenetic affiliation of Actinobacteria clade members. *Escherichia coli* ATCC 11775^T^ was used as an outgroup. **C,** Phylogenetic affiliation of Chloroflexi, Proteobacteria, and Deinococcus-Thermus clade members.

To compare the bacterial communities among these biofilms, three to five samples were collected from the top surface of each pigmented biofilm on four faces of the inner gallery (Fig. 1A) and subjected to 16S rRNA gene analyses. The numbers of DNA bands recovered from the DGGE gels were 36, 17, 24, 33, and 20 from the P, G, V, B, and BG biofilms, respectively. Of these, 24, 6, 7, 11, and 18, respectively, were successfully amplified, and then used for phylogenetic analyses after sequencing. We examined the phylogenetic relationships of these sequenced DNA bands with the DNA of members of the *Cyanobacteria* (Fig. 4A), *Actinobacteria* (Fig. 4B), and others (Fig. 4C).

Bacteria that were closely related to *Cyanobacteria* were found in many of the G, V, B, and BG biofilms, but none was found in the P biofilm (Fig. 4A). *Actinobacteria*-related bacteria prevailed in the P biofilms, especially in the genus *Rubrobacter*. In addition to the P biofilm, the G and BG biofilms included some bacteria closely related to the genus *Rubrobacter* (Fig. 4B).

*Rubrobacter*-related bacteria were observed in all pigmented biofilms collected at the Bayon and were especially rich in the P biofilm. The genus *Rubrobacter* is made up of *Rubrobacter radiotolerans*, *Rubrobacter xylanophilus*, and *Rubrobacter taiwanensis. Rubrobacter radiotolerans* has greater gamma radiation resistance than *Deinococcus radiodurans* (3, 28, 49). This radiation resistance might give *Rubrobacter*-related bacteria a growth advantage for colonizing the sandstone walls under strong sunlight at this tropical site. However, P biofilms from which the genus *Rubrobacter* were frequently detected were observed on the wall covered with a roof, suggesting colonization of uncultured bacteria that are clearly different from the *Rubrobacter* species so far known. *Rubrobacter xylanophilus* is also known to have a halotolerant phenotype. If a member of *Rubrobacter*-related bacteria possesses this trait, they will have an advantage for growing on the stone surface.

A relationship between *Rubrobacter*-related bacteria and rosy-pigmented biofilms has been reported in historical wall paintings or masonry in Europe (15, 20, 40), but to our knowledge our observation is the first of this type in Asia. Interestingly, most of the 16S rRNA sequences obtained from the P biofilm formed a cluster separate from most sequences derived from the rosy discoloration of wall paintings or masonry in Europe (15, 20, 40) (indicated by the shaded area in Fig. 4B). These results suggest that *Rubrobacter*-related bacteria colonize the rosy discoloration on historical stone monuments in Angkor Thom as well as in Europe, but their phylogenetic position differs slightly from those in Europe. Although we tried to isolate *Rubrobacter*-related bacteria by using the media described by Laiz *et al.* (20), we have not yet succeeded (data not shown).

The band sequences derived from the P, B, and BG biofilms were affiliated with members of the *Chloroflexi*, *Proteobacteria*, *Acidobacteria*, and *Deinococcus-Thermus* (Fig. 4C). Tolerance to irradiation, desiccation, and high temperature, which is known to occur in members of the phyla *Chloroflexi* and *Deinococcus-Thermus* and the genus *Rubrobacter*, appears advantageous for colonization of the sandstone walls.

**Fig. 5:**
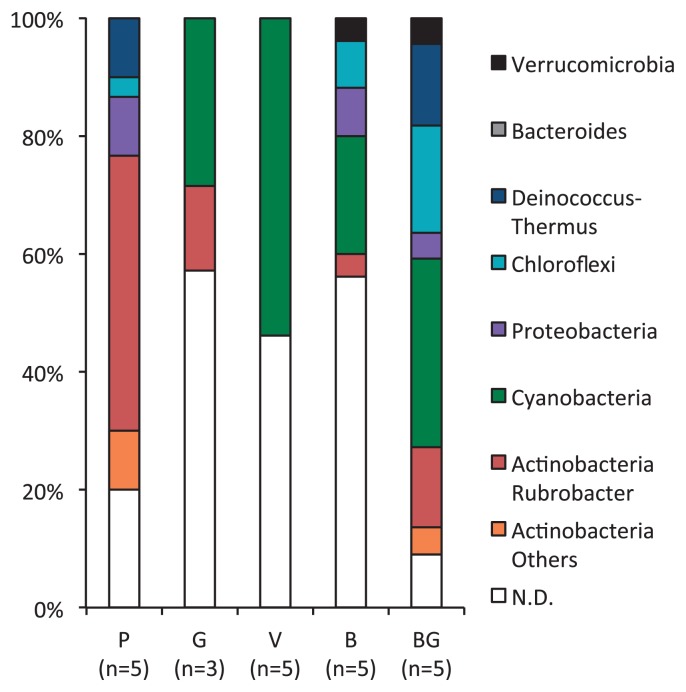
Comparison of bacterial compositions of pigmented biofilms formed on sandstone walls in Bayon. Sequences obtained from each biofilm sample were classified on the basis of the results of a BLAST search of Greengenes. Detailed affiliations are given in Table S2. P, salmon pink; B, black–gray; V, signal violet; G, chrome green; BG, blue–green; N.D., Not determined.

We calculated the bacterial compositions of each of the colored biofilms on the basis of the number of sequences obtained from the DGGE profiling followed by phylogenetic analysis (Fig. 5A). Bands at the same position in a gel were considered to represent the same taxon. Unsequenced bands were calculated as “not determined (N.D.)”. In P biofilms, members of the genus *Rubrobacter* was very rich (Fig. 5). In the V and B biofilms, bacteria belonging to the phylum *Cyanobacteria* or *Chloroflexi* had the highest species richness. In the G and BG biofilms, bacteria of the phylum *Cyanobacteria* or genus *Rubrobacter* showed high species richness. Bacteria of the phylum *Deinococcus-Thermus* were also observed in BG biofilms. In the G biofilm, although relatively few sequences were analyzed, bacteria grouped in the *Chloroflexi*, *Cyanobacteria*, and *Rubrobacter* were detected. These results suggested that the bacterial community composition of the biofilm surfaces had a considerable effect on their color. The color of the pigmentation in the biofilms could be a useful indicator of the bacterial communities present.

Members of the *Cyanobacteria* were observed in biofilms of all five colors. Some groups of *Cyanobacteria* have tolerance to extreme environmental conditions, such as radiation, desiccation, salinity, and high temperature (2, 4). This stress resistance can be advantageous for growth on sandstone walls under the severe conditions at the Bayon, as observed at a number of other historical monuments (6, 38). The *Cyanobacteria*-related bacteria detected in the biofilm samples may cause the accumulation of nitrogen nutrients in addition to organic compounds derived from photosynthesis, because some *Cyanobacteria* are known to have N_2_-fixing ability (4).

### Spatial bacterial distribution in BG biofilm

**Fig. 6:**
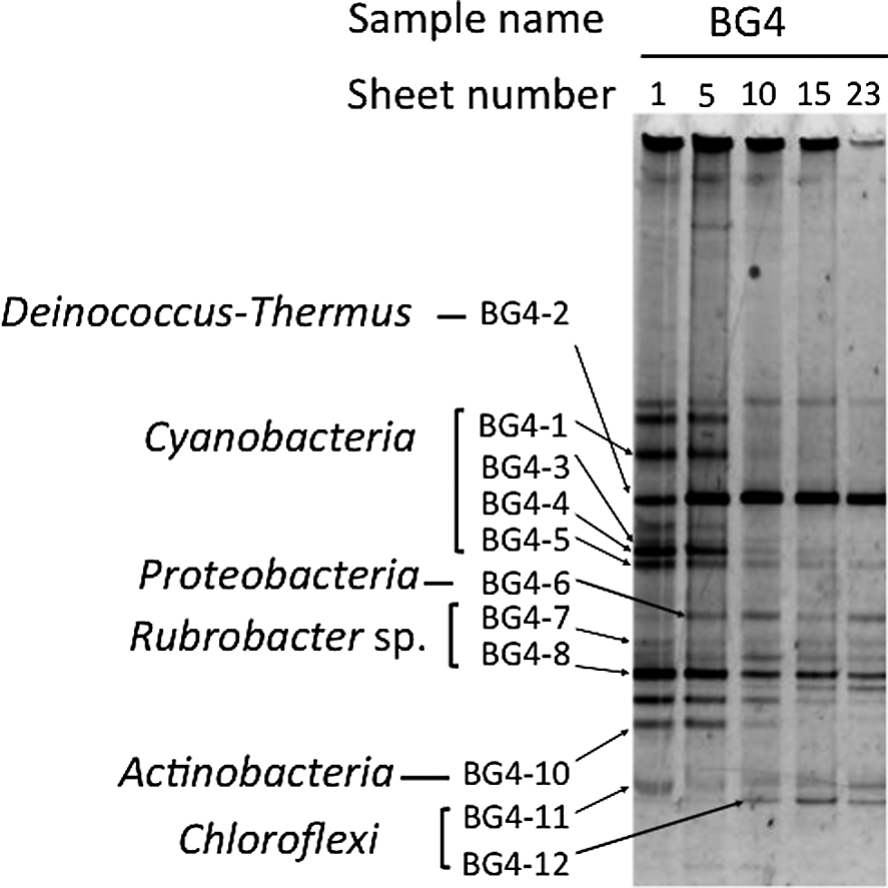
Changes in DGGE patterns of the blue–green (BG) biofilm. The sampling location, BG4, was on the south wall of a small room next to tower 26 on the south side of the inner gallery. To examine in more detail the structure of the biofilm, samples were collected from the surface to the deepest part by using 23 adhesive sheets. Phylogenetic affiliations at the phylum level are shown next to the band names. Detailed affiliations of these bands are given in Table S2.

We examined the spatial bacterial distribution in the blue–green biofilm BG4 by using the DGGE profiles of 16S rRNA gene fragments (Fig. 6). Adhesive sheets with low numbering corresponded to the surface of the biofilm and those with higher numbering corresponded to the deepest layer of the biofilm. The banding pattern changed markedly from sheet numbers 5 to 10 of the 23 adhesive sheets. There were many kinds of bacteria, especially *Cyanobacteria*-related bacteria, in the surface layer of the biofilm, but there were fewer kinds of bacteria in the deeper layer. Two predominant bacteria were present throughout the layers. One was closely related to members of the phylum *Deinococcus-Thermus*, and the other was related to members of the genus *Rubrobacter*. In addition, a *Chloroflexi*-related bacterium, derived from band BG4-12, appeared only in the deeper layer of the biofilm. These results suggested that the bacterial habitats within each biofilm are very specific depending on the availability of light and organic nutrients and the oxygen requirement.

Spatial microbial distribution has been reported, especially in aquatic biofilms in experimental culture (5, 8, 32, 42) and in some environmental biofilms (13). However, there have been few reports of the biofilms on sandstone surfaces. McNamara *et al.* (26) reported that the endolithic bacterial community in terrestrial biofilms on limestone at a Mayan site differed from the epilithic community; their findings also suggest that a stratified bacterial structure forms in the biofilms on historical monuments. Because microbes living in the deepest part of the biofilm are likely to attack the stone more directly than those that live on the surface, an understanding of stratified microbial distribution is important.

A similar layered bacterial structure has already been reported in microbial mats in a hot spring. The surface layer was made up of *Chloroflexus* (phylum *Chloroflexi*) species and *Cyanobacteria*, and the deepest layer was composed of *Roseiflexus castenholzii* (phylum *Chloroflexi*) (13). The difference in the optical absorption spectra of the *Chloroflexi* and *Cyanobacteria* might enable them to live together in this terrestrial biofilm. The phylum *Chloroflexi* contains two orders of heterotrophic bacteria: the order *Chloroflexales*, a group of filamentous anoxygenic phototrophs, and the order *Herpetosiphonales*, which consists of non-phototrophic filamentous bacteria belonging to the genus *Herpetosiphon*. We found that a number of bacteria belonging to the phylum *Chloroflexi* grew on the sandstone wall, but most of them were uncultured members. This has been confirmed in a cloning analysis of biofilms of the Bayon temple (21). Although *Chloroflexi* bacteria have been detected at a few historical sites (26, 34), most have been detected in hot springs (11, 12, 35), in hypersaline mats (30), and in freshwater (18). The role of the organisms in the biofilms formed on the Bayon remains to be elucidated, and further study is required.

### Accumulation of nitrate ions in biofilms

We examined the concentration of nitrate ions and the pH in the biofilms (Table 2). The concentration of NO_3_^−^-N in biofilm sample No. 5, which was derived from an area near samples BG1, BG4, and BG5, was 13 mg (g wet weight sample)^−1^. The concentrations of NO_3_^−^-N in the other samples derived from the roofed wall in the inner gallery ranged from 7.2 to 13 mg (g wet weight sample)^−1^. If this amount of nitrate ions were present as nitric acid, pH values would reach around 4. However, the pH values in these biofilm samples were from 6.5 to 7.5 (Table 2). This suggested that there was considerable accumulation of nitrate salts, not nitric acid, in the biofilm formed on the roofed wall. In contrast, the concentrations of nitrate ions in the biofilms where the roof were missing were in the range of 0.02 to 0.11 mg (g wet weight sample)^−1^.

The presence of a roof over the wall can prevent direct exposure to rain and direct sunlight; however, at the same time, the presence of a roof might enhance the accumulation of nitrate ions in the biofilm because the roof would prevent the washing out of nitrate ions from the wall by rainwater. The accumulated nitrate ions could be crystallized in the sandstone, which would accelerate deterioration. Both acidification and bacterial growth will damage to the stone. Therefore, greenish-colored biofilms, including the BG biofilm, likely have the potential to do more severe damage to the stone than biofilms of other colors. Future work will attempt to determine how these microbial communities in each colored biofilm correlate to sandstone deterioration. Additional work will focus on the accumulation of nitrate ions and constructive differences as well as revealing the bacterial communities in upper and lower parts of biofilm.

## Conclusions

The bacterial communities detected in the biofilms on this historical sandstone surface in Cambodia differed from those in the air. The biofilms were composed of more unknown bacteria, whereas the airborne bacteria consisted of cultured bacteria grouped mainly in the phylum *Proteobacteria* and *Actinobacteria. Rubrobacter*-related bacteria were retrieved especially from the salmon pink biofilms, whereas the black–gray, signal violet, chrome green, and blue–green biofilms were composed mainly of members of the phyla *Cyanobacteria* and *Chloroflexi*. Nitrate ion measurement suggested that the blue–green biofilm formed on the parts of the walls where the roof remained was more influential in causing stone deterioration than were the other pigmented biofilms.

## Supplementary Information



## Figures and Tables

**Table 1:** Colors and sampling locations of biofilms used for phylogenetic analysis in this study

Sample	Color of biofilm^a^	Sampling location^b^	Roof^c^
P13	Salmon pink (P)	Southwest area of tower 28	+
P20		North-facing wall between towers 35 and 36	+
P21		Northeast area of tower 36	+
P39		Southeast area of tower 24	+
P53		North-facing wall in the northwest area of tower 36	+
G01	Chrome green (G)	West-facing wall between towers 30 and 31	+
G27		South-facing wall in the southeast area of tower 37	+
G31		North-facing wall in the northeast area of tower 23	+
V11	Signal violet (V)	West-facing wall between towers 28 and 29	−
V15		South-facing wall between towers 27 and 28	−
V35		East-facing wall between towers 23 and 24	−
V37		East-facing wall between towers 23 and 24	−
V53		North-facing wall between towers 35 and 36	−
B06	Black gray (B)	West-facing wall between towers 29 and 30	−
B23		West-facing wall in the southwest area of tower 25	+
B24		East-facing wall between towers 36 and 37	+
B36		East-facing wall between towers 23 and 24	−
B53		North-facing wall between towers 35 and 36	−
BG1	Blue green (BG)		+
BG4		East-facing wall in the southeast area of tower 26	+
BG5			+

Samples P53, V53, B53, BG1, BG4, and BG5 were collected in September 2008. The other samples were collected in September 2009.

a Letters in parentheses are abbreviations of colors.

b All biofilms were obtained from the inner gallery of Bayon. Towers are numbered as described in Fig. 1A.

c +, Roof indicated in Fig. 1B was present; −, roof was missing.

**Table 2:** Nitrate ion concentration in the biofilms

Sample		Color of biofilm	Roof^a^	pH^b^	NO_3_^−^-N (mg [g wet weight sample]^−1^)
No. 5		Blue green (BG)	+	7.0	13
No. 3	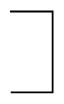	Yellow green	+	7.5	12
No. 4			+	6.5	7.2
No. 7	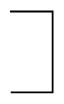	Brown beige	+	6.6	13
No. 8			+	7.0	8.2
No. 1	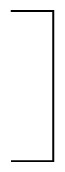	Purple violet	−	6.4	0.11
No. 2			−	7.2	0.04
No. 10			−	6.3	0.02
No. 9		Brown green	−	6.9	0.03

Samples were collected from the inner or outer gallery in September 2011.

a +, Roof indicated in Fig. 1B was present; −, roof was missing.

b Biofilms were diluted 1:4 with Milli-Q water and centrifuged; the supernatants were used for pH measurement in the field.
