# Bacterial Communities in Pigmented Biofilms Formed on the Sandstone Bas-Relief Walls of the Bayon Temple, Angkor Thom, Cambodia

**DOI:** 10.1264/jsme2.ME13033e

**Published:** 2014-03

ASAKO KUSUMI^1^, XIANSHU LI^1^, YU OSUGA^2^, ARATA KAWASHIMA^2^, JI-DONG GU^3^, MASAO NASU^4^, YOKO KATAYA MA^1,2^

^1^*United Graduate School of Agricultural Science, Tokyo University of Agriculture and Technology, 3–5–8 Saiwai-cho, Fuchu-shi, Tokyo 183–8509, Japan;* ^2^*Graduate School of Agriculture, Tokyo University of Agriculture and Technology, 3–5–8 Saiwai-cho, Fuchu-shi, Tokyo 183–8509, Japan;* ^3^*School of Biological Sciences, The University of Hong Kong, Pokfulam Road, Hong Kong SAR, People’s Republic of China; and* ^4^*Advanced Pharmaco-science, Graduate School of Pharmaceutical Sciences, Osaka University, 1–6 Yamada-oka, Suita, Osaka 565–0871, Japan*

Volume 28, no. 4, Page 422–431, 2013

Page 422

Date of “Published online” is incorrect. It should be corrected as follows:

incorrect: November 26, 2013

correct: December 13, 2013

This correction has been completed on the electronic file of the present paper available at J-STAGE.

The editorial board expresses sincere apology for the misprinting.

Microbes and Environments editorial board

