# Supplementary material for: Bacterial Communities in Pigmented Biofilms Formed on the Sandstone Bas-Relief Walls of the Bayon Temple, Angkor Thom, Cambodia
Source: Microbes Environ. 2013 Dec 13;28(4):422–31. doi: 10.1264/jsme2.ME13033 (PMC4070708; doi:10.1264/jsme2.ME13033)
Supplement: Supplementary file 1 [file 28_422_s1.pdf]

Table S1. Phylogenetic affiliations of 16S rRNA sequences retrieved in the PCR-DGGE analysis of air and biofilm samples.

Band name sequenced	Accession number	Closest BLAST match (accession number)	Identity (%)
Air			
A1	AB758608	*Pseudomonas panacis* strain CG20106 (NR_043195)	488/488 (100%)
A2	AB758609	*Phyllobacterium myrsinacearum* STM 948 (NR_043189)	437/437 (100%)
A3	AB758610	*Pseudomonas panacis* strain CG20106 (NR_043195)	506/506 (100%)
A4	AB758611	*Bosea vestrisii* 34635 (NR_028799)	390/392 (99%)
A5	AB758612	*Phyllobacterium myrsinacearum* STM 948 (NR_043189)	431/431 (100%)
A8	AB758613	*Rhodococcus erythropolis* N11 (NR_037024)	469/470 (99%)
A11	AB758614	*Pseudomonas panacis* strain CG20106 (NR_043195)	480/480 (100%)
Biofilm			
F3	AB758615	*Pseudomonas moorei* RW10 (NR_042542)	339/339 (100%)
F4	AB758616	*Rubrobacter radiotolerans* P1 (NR_029191)	384/410 (94%)
F5	AB758617	*Rubrobacter radiotolerans* P1 (NR_029191)	353/374 (94%)
F10	AB758618	*Planktothricoides raciborskii* NIES-207 (NR_040858)	354/390 (91%)
F12	AB758619	*Rubrobacter radiotolerans* P1 (NR_029191)	425/451 (94%)
F15	AB758620	*Caulobacter* sp. FWC21 (NR_041963)	247/254 (97%)
F17	AB758621	*Rubellimicrobium* sp. MSL 20 (NR_044275)	164/177 (93%)
F18	AB758622	*Rubrobacter radiotolerans* P1 (NR_029191)	275/299 (92%)

Table S2. Phylogenetic affiliation of sequences derived from DGGE analysis of biofilm samples in the Bayon.

Band name sequenced	Accession number	Closest blast match (Accession number)	Identity (%)
Salmon pink biofilm			
P13-1	AB679779	*Rubrobacter radiotolerans* strain P 1 (NR_029191)	383/409 (94%)
P13-2	AB679780	*Rubrobacter radiotolerans* strain P 1 (NR_029191)	383/410 (93%)
P13-3	AB679781	*Rubrobacter radiotolerans* strain P 1 (NR_029191)	390/415 (94%)
P20-1	AB679782	*Deinococcus peraridilitoris* KR-200 (NR_044113)	331/371 (89%)
P20-4	AB679783	*Deinococcus peraridilitoris* KR-200 (NR_044113)	363/407 (89%)
P20-5	AB679784	*Rubrobacter radiotolerans* P 1 (NR_029191)	312/343 (91%)
P21-1	AB679785	*Rubrobacter radiotolerans* P 1 (NR_029191)	390/420 (93%)
P21-4	AB679786	*Rubrobacter radiotolerans* P 1 (NR_029191)	371/400 (93%)
P21-6	AB679787	*Rubrobacter radiotolerans* P 1 (NR_029191)	372/407 (91%)
P21-10	AB679788	*Pseudonocardia chloroethenivorans* SL-1 (NR_025213)	221/234 (94%)
P21-11	AB679789	*Actinomycetospora chiangmaiensis* YIM 0006 (NR_042562)	371/405 (92%)
P39-1	AB679790	*Sphingomonas phyllosphaerae* FA2 (NR_029111)	313/331 (95%)
P39-2	AB679791	*Truepera radiovictrix* RQ-24 (NR_043482)	313/347 (90%)
P39-3	AB679792	*Sphingomonas abaci* C42 (NR_042192)	338/359 (94%)
P39-5	AB679793	*Rubrobacter radiotolerans* P 1 (NR_029191)	397/417 (95%)
P39-6	AB679794	*Rubrobacter radiotolerans* P 1 (NR_029191)	259/273 (95%)
P39-7	AB679795	*Rubrobacter taiwanensis* LS-293 (NR_025220)	384/418 (92%)

P39-8	AB679796	*Sphaerisporangium melleum* 3-28(8) (NR_041302)	186/203 (92%)
P39-10	AB679797	*Thermomicrobium roseum* (NR_044678)	321/395 (81%)
P53-1	AB679798	*Orientia tsutsugamushi* Karp (NR_025860)	173/203 (85%)
P53-4	AB679800	*Rubrobacter radiotolerans* P 1 (NR_029191)	346/375 (92%)
P53-5	AB679801	*Rubrobacter radiotolerans* P 1 (NR_029191)	312/346 (90%)
P53-6	AB679802	*Rubrobacter radiotolerans* P 1 (NR_029191)	353/384 (92%)
P53-7	AB679803	*Rubrobacter taiwanensis* LS-293 (NR_025220)	279/306 (91%)
Crome green biofilm			
G01-1	AB679824	*Planktothricoides raciborskii* NIES-207 (NR_040858)	310/335 (93%)
G01-9	AB679825	*Planktothricoides raciborskii* NIES-207 (NR_040858)	332/363 (91%)
G27-4	AB679826	*Rubidibacter lacunae* KORDI 51-2 (NR_044104)	319/364 (88%)
G27-5	AB679827	*Rubrobacter radiotolerans* P 1 (NR_029191)	496/526 (94%)
G31-1	AB679828	*Rubidibacter lacunae* KORDI 51-2 (NR_044104)	319/364 (88%)
G31-2	AB679829	*Rubrobacter radiotolerans* P 1 (NR_029191)	216/229 (94%)
Signal violet biofilm			
V15-1	AB679816	*Planktothricoides raciborskii* NIES-207 (NR_040858)	282/323 (87%)
V15-2	AB679817	*Planktothricoides raciborskii* NIES-207 (NR_040858)	287/333 (86%)
V15-3	AB679818	*Planktothricoides raciborskii* NIES-207 (NR_040858)	306/346 (88%)
V15-4	AB679819	*Planktothricoides raciborskii* NIES-207 (NR_040858)	306/349 (88%)
V53-1	AB679821	*Planktothricoides raciborskii* NIES-207 (NR_040858)	294/331 (89%)
V53-2	AB679822	*Planktothricoides raciborskii* NIES-207 (NR_040858)	212/235 (90%)

V53-4	AB679822	*Planktothricoides raciborskii* NIES-207 (NR_040858)	277/310 (89%)
Black grey biofilm			
B06-1	AB679804	*Planktothricoides raciborskii* NIES-207 (NR_040858)	311/348 (89%)
B06-2	AB679805	*Herpetosiphon geysericola* GC-42 (NR_028694)	290/339 (86%)
B23-1	AB679806	*Methylobacterium gregans* 002-074 (NR_041440)	295/360 (82%)
B23-3	AB679807	*Pseudacidovorax intermedius* CC-21 (NR_044241)	255/307 (83%)
B36-1	AB679808	*Prochlorococcus marinus* subsp. pastoris PCC 9511 (NR_028762)	336/382 (88%)
B36-2	AB679809	*Prochlorococcus marinus* subsp. pastoris PCC 9511 (NR_028762)	354/402 (88%)
B36-3	AB679810	*Prochlorococcus marinus* subsp. pastoris PCC 9511 (NR_028762)	327/371 (88%)
B36-4	AB679811	*Prochlorococcus marinus* subsp. pastoris PCC 9511 (NR_028762)	336/382 (88%)
B53-1	AB679813	*Rubrobacter radiotolerans* P 1 (NR_029191)	416/438 (95%)
B53-2	AB679814	*Chloroflexus aurantiacus* J-10-fl (NR_043411)	159/182 (87%)
B53-3	AB679815	*Verrucomicrobium spinosum* DSM 4136 (NR_026266)	178/204 (87%)
Blue green biofilm			
BG1-1	AB679757	*Truepera radiovictrix* RQ-24 (NR_043482)	223/235 (95%)
BG1-2	AB679758	*Truepera radiovictrix* RQ-24 (NR_043482)	356/392 (91%)
BG1-3	AB679759	*Rubidibacter lacunae* KORDI 51-2 (NR_044104)	249/286 (87%)
BG1-4	AB679760	*Rubidibacter lacunae* KORDI 51-2 (NR_044104)	256/288 (89%)
BG1-5	AB679761	*Salisaeta longa* S4-4 (NR_044496)	302/353 (86%)
BG1-8	AB679762	*Rubrobacter taiwanensis* LS-293 (NR_025220)	380/413 (92%)
BG1-9	AB679763	*Mycoplana dimorpha* IAM 13154 (NR_043388)	331/363 (91%)

BG1-11	AB679764	*Sphaerobacter thermophilus* DSM 20745 (NR_042118)	197/217 (91%)
BG1-12	AB679765	*Sphaerobacter thermophilus* DSM 20745 (NR_042118)	354/432 (82%)
BG4-1	AB679766	*Rubidibacter lacunae* KORDI 51-2 (NR_044104)	305/345 (88%)
BG4-2	AB679767	*Truepera radiovictrix* RQ-24 (NR_043482)	250/275 (91%)
BG4-3	AB679768	*Rubidibacter lacunae* KORDI 51-2 (NR_044104)	290/327 (89%)
BG4-4	AB679769	*Rubidibacter lacunae* KORDI 51-2 (NR_044104)	308/349 (88%)
BG4-5	AB679770	*Rubidibacter lacunae* KORDI 51-2 (NR_044104)	305/345 (88%)
BG4-6	AB679771	*Salisaeta longa* strain S4-4 (NR_044496)	298/352 (85%)
BG4-7	AB679772	*Rubrobacter taiwanensis* LS-293 (NR_025220)	291/316 (92%)
BG4-8	AB679773	*Rubrobacter taiwanensis* LS-293 (NR_025220)	337/367 (92%)
BG4-10	AB679774	*Streptomyces stramineus* NBRC 16131 (NR_041198)	224/236 (95%)
BG4-11	AB679775	*Sphaerobacter thermophilus* DSM 20745 (NR_042118)	249/274 (91%)
BG4-12	AB679776	*Sphaerobacter thermophilus* DSM 20745 (NR_042118)	202/222 (91%)
BG5-1	AB679777	*Roseibacillus persicicus* YM26-010 (NR_041623)	259/310 (84%)
BG5-6	AB679778	*Rubidibacter lacunae* KORDI 51-2 (NR_044104)	290/327 (89%)

4
